# The effect of Zika virus infection in the ferret

**DOI:** 10.1002/cne.24640

**Published:** 2019-02-15

**Authors:** Elizabeth B. Hutchinson, Mitali Chatterjee, Laura Reyes, Francis T. Djankpa, William G. Valiant, Bernard Dardzinski, Joseph J. Mattapallil, Carlo Pierpaoli, Sharon L. Juliano

**Affiliations:** ^1^ Program in Neuroscience USUHS Bethesda Maryland; ^2^ Department of Anatomy Physiology and Genetics Bethesda Maryland; ^3^ Department of Microbiology and Immunology Bethesda Maryland; ^4^ Program in Emerging and Infectious Disease Bethesda Maryland; ^5^ Department of Radiology Bethesda Maryland; ^6^ Quantitative Medical Imaging Section National Institute of Biomedical Imaging and Bioengineering, National Institutes of Health Bethesda Maryland

**Keywords:** CT, diffusion tensor MRI, microcephaly, neural development, neural progenitor, RRID:AB_11217435, RRID:AB_234119, RRID:AB_609914, RRID:AB_726362, vasculature

## Abstract

Although initial observations of infections with the Zika virus describe a mild illness, more recent reports show that infections by Zika result in neurotropism. In 2015, substantial congenital malformations were observed, with numerous infants born with microcephaly in Brazil. To study the underlying mechanism and effects of the disease, it is critical to find suitable animal models. Rodents lack an immune system parallel to humans and also have lissencephalic brains, which are likely to react differently to infections. As the smallest gyrencephalic mammal, ferrets may provide an important animal model to study the Zika virus, as their brains share many characteristics with humans. To evaluate the prospect of using ferrets to study Zika virus infection, we injected seven pregnant jills with the PR strain subcutaneously on gestational day 21, corresponding to the initiation of corticogenesis. These injections resulted in mixed effects. Two animals died of apparent infection, and all kits were resorbed in another animal that did not die. The other four animals remained pregnant until gestational day 40, when the kits were delivered by caesarian section. We evaluated the animals using CT, MRI, diffusion tensor imaging, and immunohistochemistry. The kits displayed a number of features compatible with an infection that impacted both the brain and skull. The outcomes, however, were variable and differed within and across litters, which ranged from the absence of observable abnormalities to prominent changes, suggesting differential vulnerability of kits to infection by the Zika virus or to subsequent mechanisms of neurodevelopmental disruption.

## 1 INTRODUCTION

As evidence appeared that the Zika virus affects the brain during development and can result in severe neurologic impairments and anatomic malformations, a targeted research effort was launched to understand the underlying neurobiological factors contributing to this problem. Although recent studies clarify many aspects of Zika infection, countless questions remain. An appropriate animal model would be an important avenue for answering persistent mechanistic questions, despite several years of a concerted effort to understand the timing, route of transmission, and specificity of the Zika virus. Although rodents have been highly useful in beginning to clarify information about Zika infection, in order to demonstrate neurologic effects of the virus it is usually necessary to use immunoincompetant mice, to block antibody production, or to provide direct infection into the brain (e.g., Li, Saucedo‐Cuevas, Shresta, & Gleeson, 2016; Shao et al., 2016; Smith, 2017). Additionally, the development of the lissencephalic mouse brain has several key differences from human cortical development that limit the translation of findings for this application. Thus, the development of complementary animal models to study Zika infection and the outcomes are essential to address the current limitations of rodent models.

The ferret is an interesting and potentially translational animal model to study the effects of Zika virus. Ferrets are the smallest mammal with a gyrencephalic cerebral cortex and posess several key features and an immune system that make them appropriate for study of neurotropy evoked by this virus (Barnard, 2009; Empie, Rangarajan, & Juul, 2015). In fact, models of microcephaly have been developed in ferrets in response to environmental toxins delivered during gestation, which cause the brains of the offspring to be smaller and reduced in overall neocortical folding (Poluch & Juliano, 2015). These findings are important to demonstrate that the ferret is susceptible to microcephaly.

Ferrets have also been used for many years to study viruses that affect people and are the predominant animal species for study of influenza based on wet transmission of virus and human‐similar immune response (Albrecht et al., 2018; Belser, Eckert, Tumpey, & Maines, 2016; Enkirch & von Messling, 2015; Kiseleva et al., 2018). However, susceptibility of the ferret to flaviviruses—the family of viruses that includes Zika, and also viruses that cause Dengue and Yellow fever, has yet to be concluded. The Zika virus consists of a single strand RNA that encodes three structural and seven nonstructural proteins. It surfaced originally in the Zika forest of Uganda using a sentinel monkey. In the 1970s, people in several African countries displayed relatively mild symptoms including headache, fever, and joint pains after infection with Zika. The *Aedes egypti* mosquito was identified as the carrier and infection with Zika appeared as a relatively mild illness restricted to the African continent. In 2007, a breakout in Micronesia demonstrated that the virus could spread out of Africa. The initial evidence of neurotropism occurred in 2013 when individuals infected with Zika developed Guillain Barre syndrome. The first case of microcephaly was observed in Brazil a few years later.

It would be useful to understand a number of factors regarding the etiology and specificity of this problem. Although evidence is accumulating that earlier is worse than later, we still do not know exactly when during pregnancy the Zika virus elicits microcephaly; we do not know the precise route of infection; we do not know if other more subtle effects occur in infants without obvious deficits at birth. We also do not understand why only a fraction of infants born to infected mothers appear to show abnormalities. Additionally not clear is why the incidence of severe neurologic problems after Zika infection appears to be declining.

Given the morphometric nature of cranial and cerebral abnormalities associated with Zika infection, noninvasive imaging of the skull and brain can provide powerful outcome measures for characterizing the effects of Zika infection in experimental models. Diffusion MRI based methods, such as diffusion tensor imaging (DTI), can report alterations in the microscale tissue environment that correspond to abnormalities at the level of cells and vascular structures.

The objective of this study was to determine the effects of gestational Zika infection on cerebral morphometry, vascular integrity, and corticogenesis in ferrets. Abnormalities were identified and characterized using multiple complementary methods including gross body dimension measurements, targeted immunohistochemistry and noninvasive imaging of the skull by CT and of the brain by MRI. Advanced MRI analysis using morphometric and diffusion techniques has been previously used for the ferret brain during development (Knutsen, Kroenke, Chang, Taber, & Bayly, 2013; Kroenke, Taber, Leigland, Knutsen, & Bayly, 2009) and in the adult (Hutchinson et al., 2017). While research of the Zika virus remains a nascent field in many ways, this study provides a unique perspective by identifying several types of brain abnormalities caused by Zika infection in a human‐relevant species.

## 2 MATERIALS AND METHODS

### 2.1 Animals and infection with Zika virus

A total of 10 pregnant ferrets (*Mustella putoris furo*) were used in this study. Timed pregnant animals were purchased from Marshall Farms (New Rose, NY). All procedures were carried out with the approval of the USU animal care and use committee and in accordance with the US Public Health Service's Policy on Humane Care and Use of Laboratory Animals, and the Guide for the Care and Use of Laboratory Animals.

**Figure 1:**
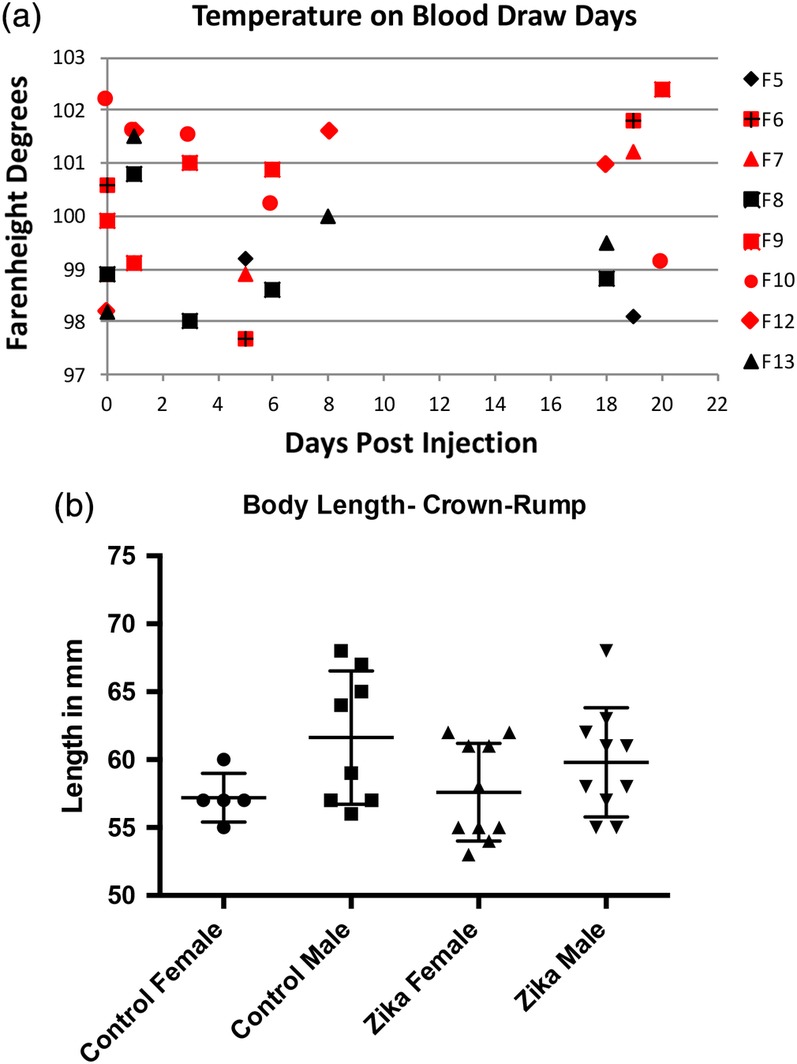
(a) This graph shows the temperature of each animal on days that we drew blood. Animals with red symbols received injections of Zika virus, while those with black symbols are controls. Although the infected animals seem to show higher overall temperatures, there were no significant differences. (b) Graph of the size (crown‐rump length) of control and Zika treated ferret kits. Included in the graph are measurements of 4 litters, 2 control, and 2 Zika treated. These measurements show that the overall size of the kits is variable. There were no significant differences between groups

The ferrets were injected subcutaneously with the Zika virus on gestational Day 20 (E20). Seven pregnant ferrets received 10^6^ TCID_50_/ml of the Puerto Rican strain of the virus (Genbank KU501215); three control jills received an injection with vehicle. The Puerto Rican strain is noted for being similar to the strains involved in recent epidemics (Quicke et al., 2016). Plasma samples were obtained on the days shown in Figure 1a. Temperatures of the pregnant animals were also obtained.

### 2.2 Absolute quantification of plasma viral loads by qRT‐PCR

Plasma viral loads were quantified as described previously (George et al., 2017). Briefly, we extracted RNA from the plasma using the QIAamp MinElute Virus spin kit (Qiagen, Germantown, MD, USA) and reverse transcribed using a mixture of random hexamers and anchored oligo‐dT primers. We amplified synthesized cDNA for PCR using Zika specific primers and probes (forward: GGAAAAAAGAGGCTATGGAAATAATAAAG; reverse: CTCCTTCCTAGCATTGATTATTCTCA; probe: AGTTCAAGAAAGATCTGGCTG) (Abbink et al., 2016). The PCR reaction was set up in triplicate for each sample using Taq‐polymerase (Bioline, Inc., Taunton, MA) and assayed in the 7,500 Taqman instrument (Applied Biosystems) using the following conditions: 48°C for 30 min, 95°C for 10 min followed by 40 cycles of 95°C for 15 s and 1 min at 60°C. The absolute number of Zika viral copies was determined using Zika virus specific standards. The limit of detection was 50 copies/ml.

### 2.3 Tissue processing and imaging

Ferret kits are born after 41 days of gestation; on embryonic day 40 we performed cesarean sections under anesthesia (5% isofluorane) and the kits removed from the mother. No complications accompanied any of the control pregnancies, but the pregnant infected animals included one found with all the kits resorbed, one that gave birth on the day scheduled for the cesarean section but found dead (the kits were saved) and one that died on embryonic day 34 (E34); the kits were harvested but not analyzed for this study. This led to 22 kits obtained from control animals and 51 kits taken from jills injected with the Zika virus.

Blood samples were taken under anesthesia (5% isofluorane) from the pregnant animals just prior to infection and on several days following the subcutaneous injection (see Figure 1a). Organs were removed for analysis and frozen, including pieces of the heart, spleen, eye, kidney, liver, and lung of the jills. After harvesting the organs, all kits were placed in fixative (4% buffered paraformaldehyde) and saved for various analyses.

Selected kits were imaged using computed tomography (CT) and/or magnetic resonance imaging (MRI). CT images were acquired on a Bruker SkyScan 1,278 (Kontich, Belgium) in vivo micro CT scanner with 52 μm isotropic resolution, 49 kV and 1 ma source voltage and current, respectively, using a 0.5 mm aluminum filter. The total acquisition time was 4 m:25 s. Reconstructed images were imported from the Tanslational Imaging Facilty rand saved into Neuroimaging Informatics Technology Initiative (NIfTI) format for further visualization.

Ex vivo MRI and diffusion tensor MRI (DTI) was performed for 15 specimens grouped into two cohorts. MRI cohort 1 (*n* = 8) included brains from different litters with Zika injection as well as from control ferret kits; MRI scans were acquired with high‐resolution over approximately 40 hr for each specimen. MRI cohort 2 included eight specimens from a single Zika‐injected litter (taken on E40) in order to determine the heterogeneity of MRI abnormalities within the same litter. The MRI scans for each specimen in this group were acquired with less scan time (approximately 24 hr) and with lower resolution. The MRI and DTI acquisition protocols for each cohort were:

#### 2.3.1 Protocol for MRI cohort 1

A high‐resolution 3D gradient echo pulse sequence acquired 50 μm isotropic T1W MRI volumes from each specimen with TE/TR = 35/60 ms, flip angle = 22°, nex = 8. Multi‐slice‐multi‐echo (MSME) T2 weighted (T2W) images were acquired using a 3D spin echo pulse sequence with TE = 10–100 ms, TR = 2000 ms, nex = 1. The spatial dimensions of this scan resulted in 150 μm isotropic voxel dimensions. For DTI, 112 DWIs were collected with the diffusion encoding scheme of b/#dirs100/6, 200/6, 500/6, 1,000/6, 1,500/32 and 4,500/56. A 3D EPI pulse sequence was used for this acquisition with TE/TR = 40/700 ms, nex = 1, segments = 8 and isotropic voxel resolution of 100 μm. One repetition was collected for each diffusion weighted image (DWI) although a subset was acquired again with opposite phase encode direction for use with geometric distortion correction.

#### 2.3.2 Protocol for MRI cohort 2

A similar T2 MSME acquisition as above was used, but with lower spatial resolution 200 μm isotropic and a shorter TR of 2,000 ms. The DTI acquisition was also similar for this cohort except with reduced resolution of 150 μm isotropic and without the lowest (b = 100 s/mm^2^) shell.

Images were imported and processed using TORTOISE3 (Irfanoglu, Nayak, Jenkins, & Pierpaoli, 2017; Pierpaoli et al., 2010) and ANTs (Avants, Epstein, Grossman, & Gee, 2008) software tools. High resolution MRI scans from protocol 1 were rigidly aligned with each other for side‐by‐side comparison of contrast features and size. To aid in this comparison, head and brain masks were generated for each of the specimens using the semi‐automatic regions growing tool of the ITKsnap software (Yushkevich et al., 2009, www.itksnap.org). The brain mask from one of the control specimens was used as an outline overlay to visually compare gross volumetric and anatomic abnormalities across specimens.

For DTI from both protocols, DWI volumes were first corrected for apparent motion artifacts and geometric distortions using the DIFFPREP and DRBUDDI modules of TORTOISE3 along with the T2W image as a registration target (Irfanoglu et al., 2015). DTI scalar maps were generated including the Trace, fractional anisotropy (FA) (Pierpaoli, Jezzard, Basser, Barnett, & Di Chiro, 1996), linear anisotropy and planar anisotropy (Westin et al., 2002) and directionally encoded color (DEC) maps (Pajevic & Pierpaoli, 1999). These maps were qualitatively inspected and also used for quantitative voxelwise analysis of DTI values and morphometry. To accomplish the latter analysis, advanced DTI‐based registration of DT volumes first generated study‐specific templates of the control or Zika‐negative groups from each cohort and then registered every individual brain of each cohort to the template (Irfanoglu et al., 2016). From these registered images, voxelwise comparisons of DTI values were made using subtraction or effect size maps of Cohen's D, which is the subtraction of the control group average from the Zika group average divided by the standard deviation of the control group. The inverse warping of each brain to the template space was used to warp a single template ROI mask of the whole brain into the native space of each individual to provide a consistent measurement of brain volume across specimens. These values were subject to statistical analysis using the Mann–Whitney *U*‐test. Finally, DTI‐driven tensor based morphometry was performed to generate LogJ maps reporting local volume differences between each brain and the template, where LogJ is the tensor based morphometry (TBM) metric of the log of the determinant of the Jacobian of the deformations field (Ashburner & Friston, 2001; Davatzikos et al., 1996; Sadeghi et al., 2018) and is positive/negative when a voxel is larger/smaller in the warped brain than the template. The LogJ maps were also used for voxelwise analysis of effect size.

### 2.4 Immunohistochemisty

After the kits remained in fixative for at least 1 week, selected brains were cut in the coronal plane using a vibratome at 50 μm thickness. Brain sections were immunoreacted by hydration in PBS, followed by blocking with a buffer comprised of 5% normal goat serum, 2% bovine serum albumin, 0.1% triton X‐100 for 1 hr. We then incubated with the primary antibody (see Table 1 for antibody information) overnight at 4°C. We washed each section several times with PBS, followed by immersion in the appropriate Alexa‐Flour secondary antibody (Invitrogen, Grand Island, MO). For each antibody, sections were run without the primary antibody to insure specificity. If the sections were double‐labeled, the primary antibodies were incubated together. We obtained images of the labeling using a Zeiss Axio Observer.Z1 microscope with an Apotome using Zen 2012 software (Blue edition) version 1.1.2.0.

**Table 1:** Antibody information

Primary antibody name	Company	Catalog #	Monoclonal polyclonal	Species it was raised in	Concentration	Secondary antibody	Catalog #	Company	Concentration	PRID
Anti vimentin Produced from pig V9 clone, an IgG isotype; recognizes ~58 kDa protein.	Sigma	V6389	Monoclonal	Mouse	1:200	Goat antimouse IgG (H + L), Alexa flour 555	A21422	Thermo fisher	1:500	Sigma‐Aldrich cat# V6389, RRID:AB_609914
Anti AXL Detects total levels of AXL protein and does not cross react with closely related intermediate filament proteins such as desmin & GFAP.	Cell signaling	C89E7	Monoclonal	Rabbit	1:100	Goat antirabbit IgG (H + L), Alexa flour 488	A11008	Thermo fisher	1:500	Cell signaling technology cat# 8661S, RRID:AB_11217435
Anti SOX2 Derived from residues ranging from 300 to the C terminus of human Sox2.	Abcam	ab97959	Polyclonal	Rabbit	1:200	Goat antirabbit IgG (H + L), Alexa flour 488	A11008	Thermo fisher	1:500	Abcam cat# ab97959, RRID:AB_234119
Anti CD31 Raised within aa 700 to the C‐terminus.	Abcam	ab28364	Polyclonal	Rabbit	1:15	Goat antirabbit IgG (H + L), Alexa flour 555	A21428	Thermo fisher	1:500	Abcam cat# ab28364, RRID:AB_726362

## 3 RESULTS

### 3.1 Blood and organ testing using PCR

Our analysis of plasma and tissue samples using RT‐PCR did not find the presence of viral load at any of the time points we examined.

### 3.2 Effects of injection

As indicated in the Materials and Methods, seven pregnant animals were injected with the Zika virus, three were additional controls. Of the seven infected, one died on embryonic Day 34; the kits were saved but not analyzed extensively. A different ferret died on the day of the scheduled cesarean section (E40), but the kits were delivered prior to her death and saved for analysis. In an additional infected pregnant ferret, all the kits were resorbed. The four remaining animals received a cesarean section on E40. This produced 22 kits obtained from the three control animals and 51 kits taken from the four surviving jills and the animal that died on E40, which were each injected with the Zika virus (total injected jills producing kits, *n* = 5).

### 3.3 Infection with Zika virus shows variability in the offspring

**Figure 2:**
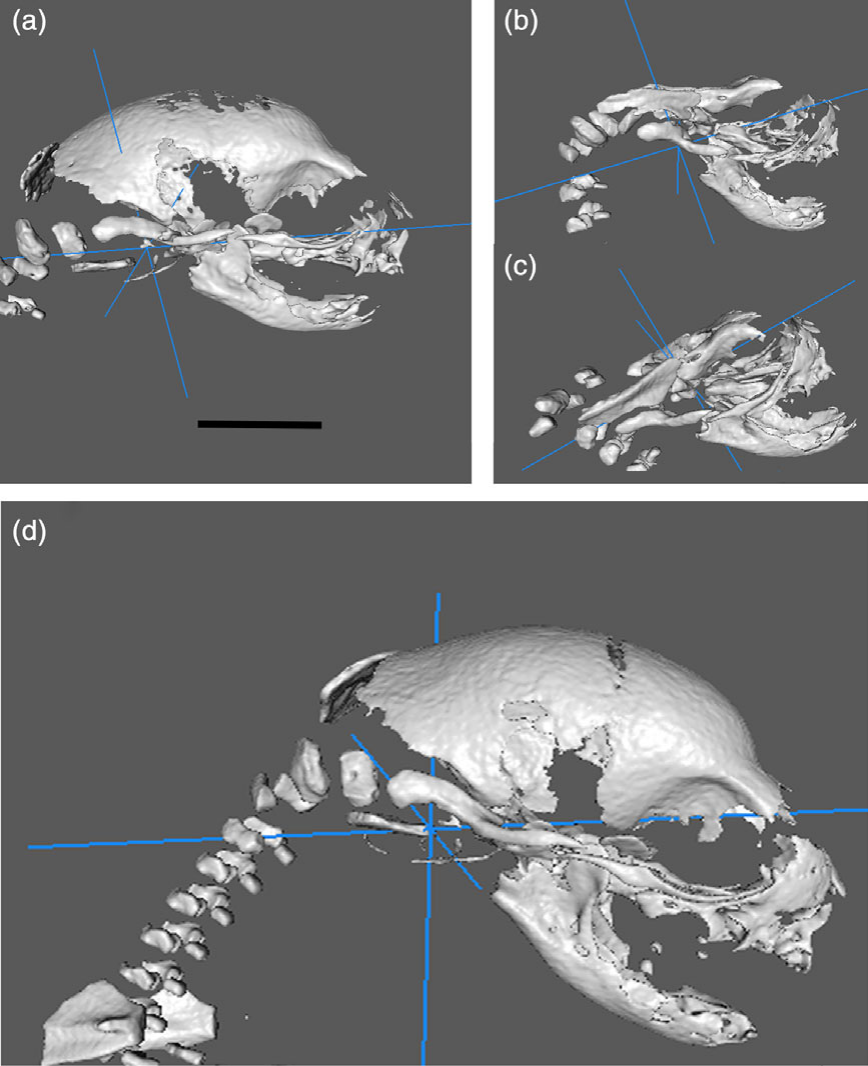
Examples of CT reconstructions taken from the same litter (a–c) infected with the Zika virus and from a control animal (d). These demonstrate that substantial variability of skull size in the same litter occurs after virus infection. The scale equals 5 mm

When we viewed the kits in a given litter, our first observation disclosed substantial variability in the size and morphology of the kits and their skulls. Figure 1b shows that substantial variability occurs in the size (body length) of both the control and Zika treated animals. The temperatures are also variable, but generally higher for the infected animals (Figure 1a). Figure 2, however, displays examples of CT reconstructions of the skulls of three kits from a single litter whose mother received an injection of the Zika virus subcutaneously at embryonic Day 20 (E20). It should be noted that E20 is the approximate date that the laminar formation of the cerebral cortex begins in central regions of the ferret, that is, somatomotor (Noctor, Scholnicoff, & Juliano, 1997). It is obvious that great variability exists between the members of the litter when compared with each other and the skull of a control animal (Figure 2). Two skulls show dramatic alterations in morphology (Figure 2b,c), while others show less extensive changes, and one skull appears relatively normal (Figure 2a) but smaller than the control skull (Figure 2a). In other litters, we also found considerable variability with the skulls and often several animals were substantially smaller than the others. These observations led us to conclude that the effects of infection with the Zika virus were highly variable within a given litter.

As we did not find a viral load in the plasma samples we collected or in the organs, including the heart, lung, spleen, and kidney, in an attempt to better clarify the presence or absence of Zika infection in a litter given the obvious deformities as seen in Figure 2, we need to find a method of separating the kits into those with apparent abnormality and those that were relatively normal. In that regard we evaluated three litters injected with Zika virus and an additional control litter using CT and/or MRI. The CT analysis led us to observe that in the infected cohort, but not the control, several animals showed deformed skulls (e.g., Figure 2), or skulls much smaller than others in the group. This can be seen in Supporting Information Figures S1 and S2. Supporting Information Figure S1 shows CT reconstructions in a movie format of skulls from a control animal; these images show relative similarity in size. Supporting Information Figure S2 shows reconstructions where the pregnant ferret received Zika virus during gestation. There is substantial variability in the size of the skulls in Supporting Information Figure S2, compared with those in Supporting Information Figure S1, where the pregnant ferret did not receive a viral injection.

Furthermore, the MRI analyses revealed that the brains showed additional deformities, which are described below.

## 4 MRI

**Figure 3:**
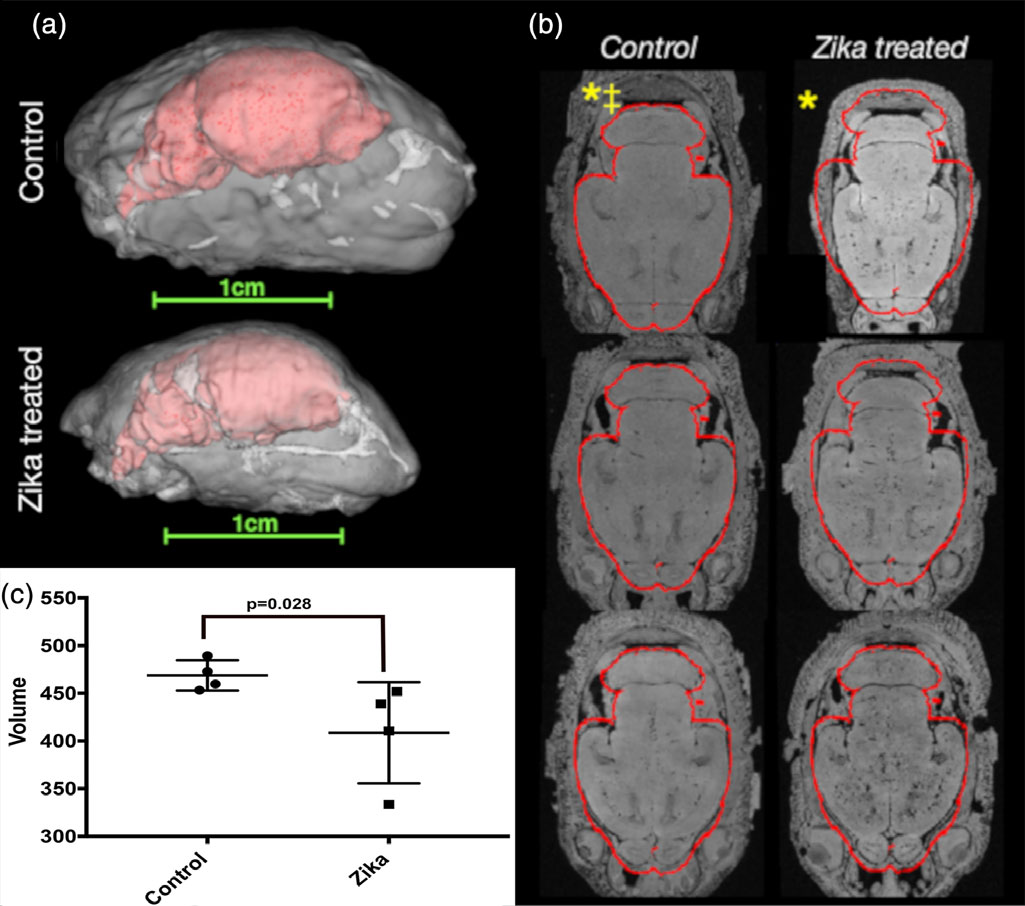
Gross anatomy and volume abnormalities in Zika treated ferret brains (MRI cohort 1). 3D reconstructions of segmented ROI masks for the brains (red) within the heads (white) of ferrets at P0 are shown (a) with the same scaling to demonstrate gross differences in size and anatomy between control and Zika treated specimens. High‐resolution axial images with the same alignment and slice level are shown for all P0 brains in the first imaging cohort of this study (b). A red outline is shown based on the mask for a single control brain and little deviation of the control brain volumes from this outline was found, while the Zika brains were found to be smaller. * denotes brains from b shown in (a). ‡ denotes control brain segmentation used to generate brain mask outline. (c) Is a plot of MRI obtained brain volumes (in mm^3^) that shows the overall volume of the Zika‐treated brains are significantly smaller than the control brains (*p* < 0.028, Mann–Whitney *U* test)

Using MRI allowed us to assess volumetric and morphologic features of Zika treated animals as compared with controls. Figure 3a shows examples of MRI‐based head and brain volumes of control and Zika treated animals with the same spatial scaling to demonstrate gross anatomical differences—especially volume reduction—of the brains and heads of animals born from infected jills. Similar gross anatomic differences were found for all Zika treated ferrets and none of the uninfected brains (Figure 3b). Quantitative template‐based ROI analysis for whole‐brain brain volumes confirmed these observations with a 12.8% mean reduction in brain volume for the group ranging between 3.6 and 28.8% reduced volume for individual brains. Despite the small sample size for this cohort there was a statistically significant difference (*p* = 0.028, Mann–Whitney *U*‐test) in whole brain volume between Zika treated and control brains (Figure 3c).

**Figure 4:**
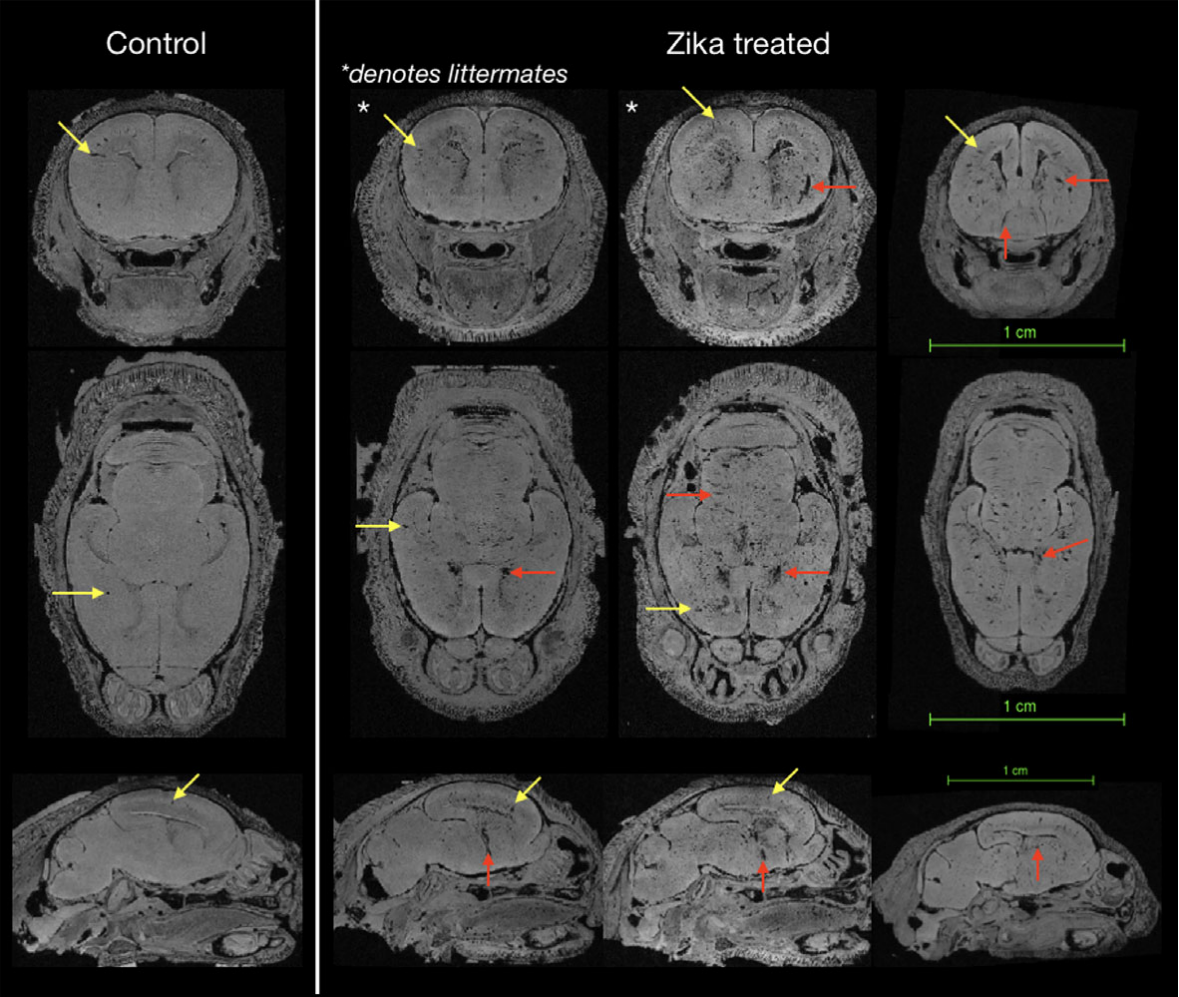
High‐resolution MRI to identify varicosities of control brains and those in Zika treated animals. Axial slices at the same level are shown for four heads after rigid alignment and regions of hypointensity were identified as varicosities (red and yellow arrows). The control brain shows the expected level of varicosities, while the examples to the right shows two litter mates with different levels of hypointensities (asterisks) and another brain from a different litter. The littermate to the left shows a brain with a lower number of brain varicosities, while the animal to the right reveals a substantially greater number of hypointensities. The yellow arrows show examples of hypointensities in all brains, while the red arrows show examples of abnormally appearing varicosities

In addition to volumetric findings, several observations were also made using MRI and DTI values in the brain. First, T1‐weighted hypointensities were conspicuous for all brains in this study and expected to correspond to susceptibility artifacts from blood in the cerebral vasculature as the specimens were not perfused to remove blood. There was, however, variability in this type of abnormality within the same Zika treated litter. In the control specimens, the hypointensities were consistent with normal vascular anatomy (see yellow arrows, Figure 4), while in the Zika treated litters we also found variability. We observed brains with fewer apparent abnormalities showing a smaller number of hypointensities (Figure 4), while other litter mates had brain regions with a higher amount of hypointensities (see red arrows, Figure 4).

**Figure 5:**
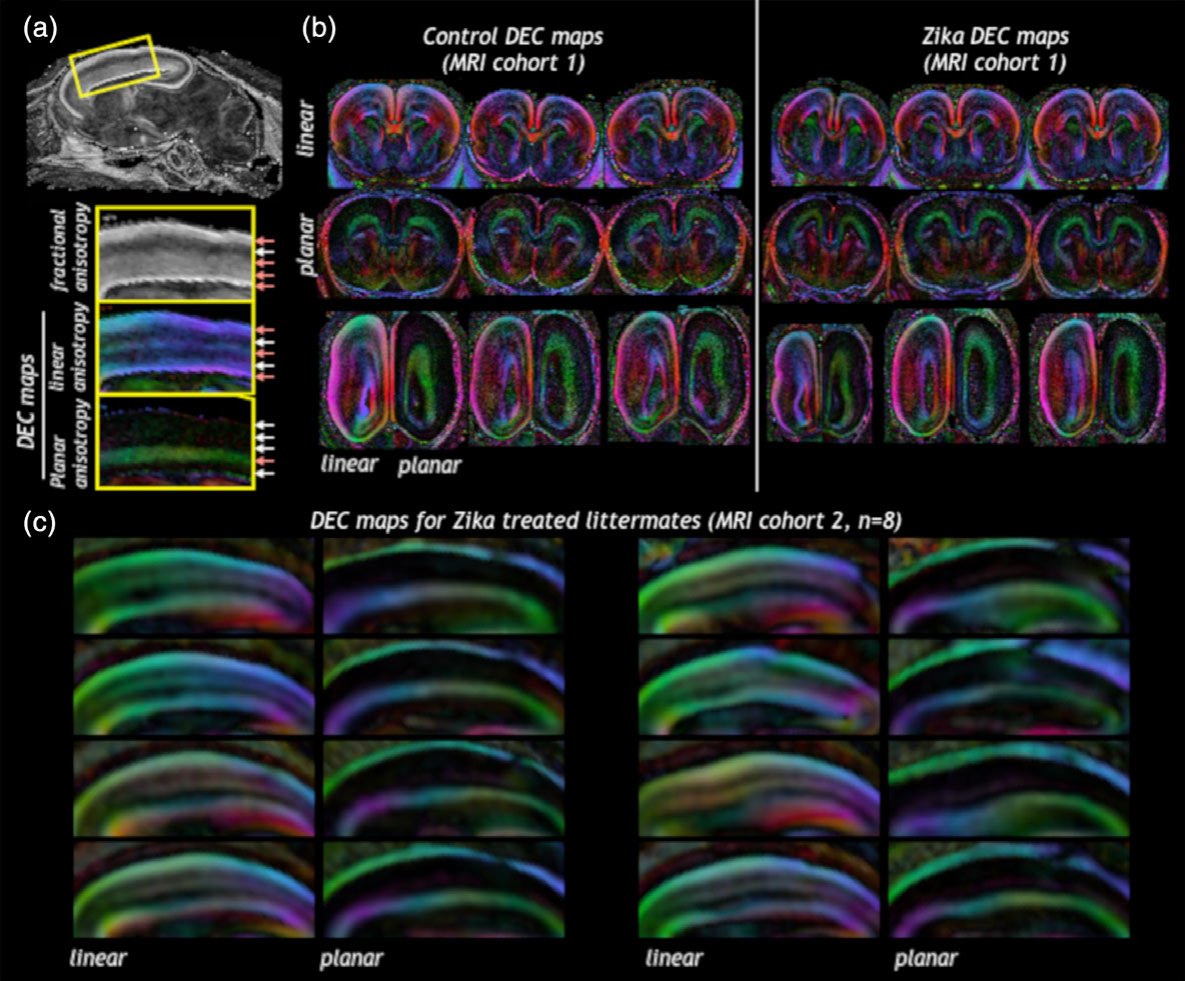
Cortical zones maintain their overall regional organization according to DTI features in control or Zika treated brains. Linear and planar anisotropy weighted directionally encoded color (DEC) maps were able to distinguish between different cortical zones (a) according to intensity and color of the metric. The presence and organization of cortical zones as indicated by DEC maps were similar in brains from Zika infected litters as from controls (b, MRI cohort 1 and c, MRI cohort 2). Note: This analysis measured laminar organization only, for volume analysis see Figure 7b

Scalar DTI values in the brain also determined the presence of neuroanatomic or microstructural abnormalities. Because the gestational period of infection is a time of robust cortical development, we visualized the organization of cortical zones that support neurogenesis and migration using DTI‐based anisotropy measures of linear and planar anisotropy, which delineate adjacent zones based on the shape of water diffusion (Figure 5a). All brains from the control and Zika‐treated groups in both cohorts demonstrated the similar laminar patterns of anisotropy (Figure 5b,c) suggesting that the presence and organization of cortical zones related to neurogenesis and migration were not severely disorganized in the Zika‐treated ferrets. The Zika treated brains were overall smaller, however, and the dimension of these zones diminished in the affected animals.

**Figure 6:**
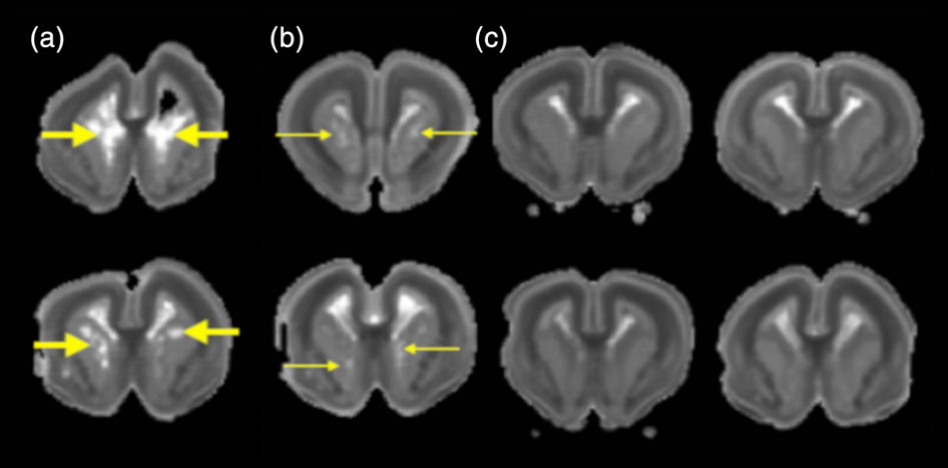
Voxelwise diffusivity abnormalities of the GE. (a) Trace maps for individual brains from MRI cohort 2 reveal prominently increased trace lesions in the GE (thick yellow arrows) for 2/8 of the brains, (b) while visible trace lesions appear for an additional 2/8 brains (thin yellow arrows) and no lesions evident for the remaining brains of this cohort (c). These trace values suggest vulnerability of the GE with variability across Zika treated littermates

Within the cohort of Zika infected littermates, increased diffusivity in the ventricular zone of the ganglionic eminence (GE) was observed for 4/8 brains. In two cases, these focal abnormalities were remarkably prominent (Figure 6a), and for the other two cases the lesions were mildly apparent, to a substantially lesser degree (Figure 6b). The remainder of brains in this cohort showed no abnormalities in the GE.

**Figure 7:**
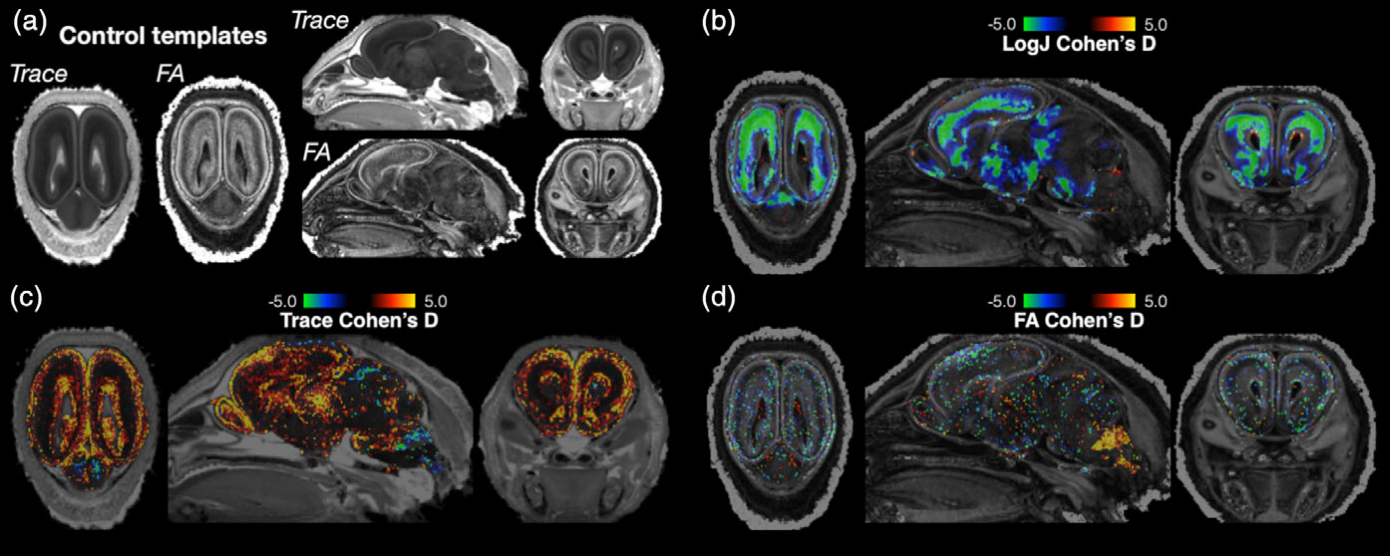
Voxelwise DTI and DTBM analysis of ferret brains. Brains from ferrets not infected with Zika virus were used to generate study specific DTI templates (a) and individual brain DTI volumes from Zika infected litters were warped to the template in order to calculate voxelwise effect size maps of local volume change (LogJ, b), diffusivity (trace, c), and anisotropy (FA, d). Cohen's D maps for LogJ—A morphometric measure—Showed prominent neocortical regions of decreased local volume for the Zika infected brains. This is especially evident by negative Cohen's D values (green color) in b

Voxelwise DTI and DTBM analyses were performed to identify microstructural and morphometric abnormalities in the Zika infected litters (Figure 7). Effect size maps for LogJ showed pronounced morphometric reductions in the neocortex especially in the intermediate zone and outer subventricular zone. Increased Trace was observed by effect size maps especially for the neocortex in regions of the cortical plate and ventricular zone. FA changes were not evident on the effect size maps.

We used the MRI and DTI abnormalities to select brains within our study for further analysis and to target particular pathology for investigation using immunohistochemitry. Vascular abnormalities were specifically expected based on the observed hypointensities throughout the brain (Figure 4). The proliferative zones of the cortex were hypothesized at the outset of the study to demonstrate abnormalities, substantiated by the MRI findings especially for examining reduced local volume of selected neocortical zones as apparent in the DTBM analysis (Figure 7b). The GE was also targeted for additional analysis based on the observed Trace lesions (Figure 6).

## 5 BLOOD VESSELS

**Figure 8:**
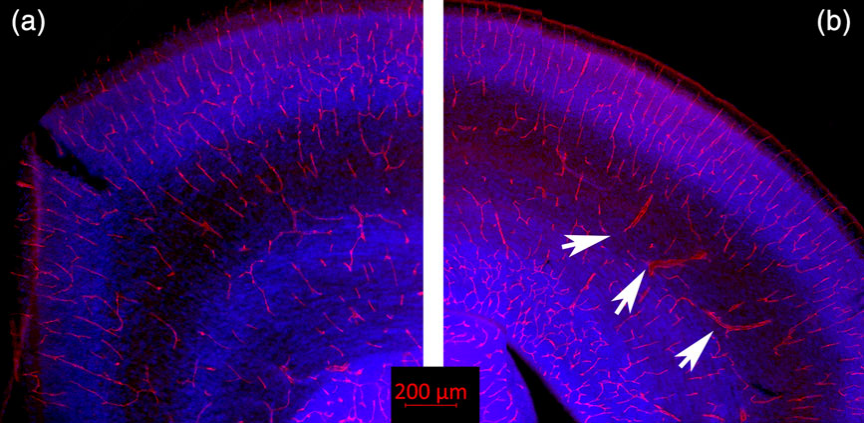
Blood vessel immunoreactivity. (a) Shown here is a normal ferret brain at P0 immunoreacted for CD31 (red), which visualizes the endothelial cells lining blood vessels and on platelets. The blue color shows cell nuclei (Dapi). (b) Shows a tissue section taken from an animal infected with the Zika virus, also immunoreacted with CD31 and stained with Dapi. The Zika treated tissue shows multiple enlarged blood vessels with an abnormal morphology (several are indicated with white arrows)

**Figure 9:**
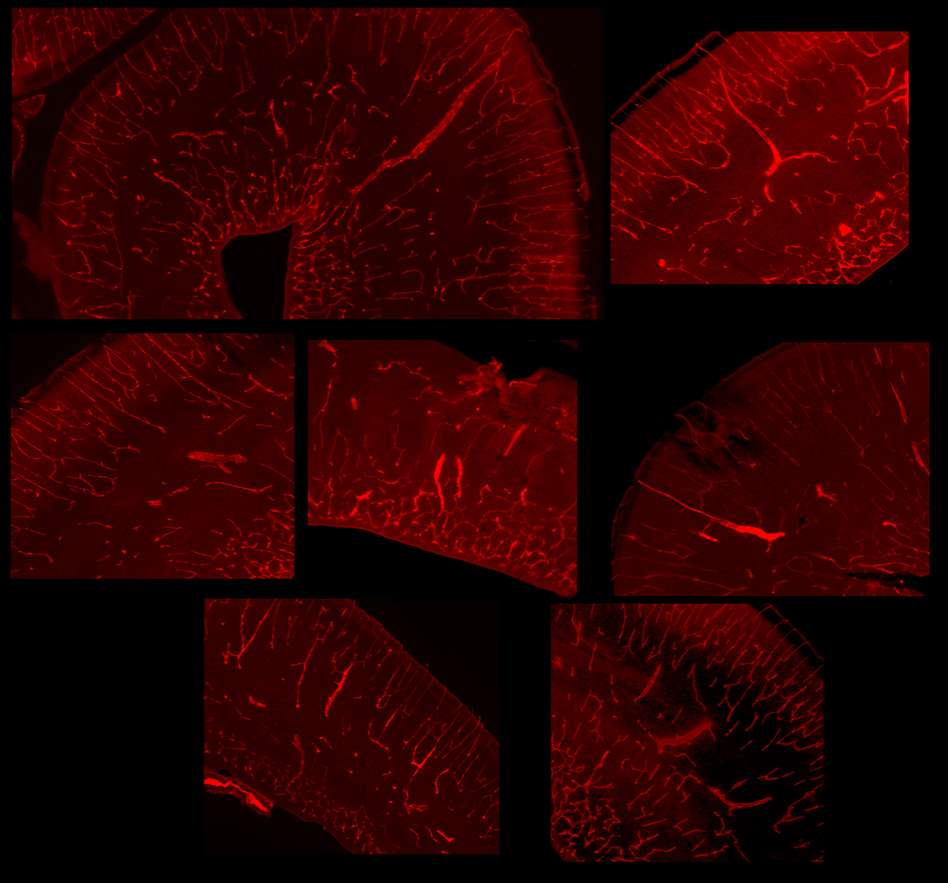
Additional images of tissue sections immunoreacted against CD31 to reveal blood vessels taken from Zika treated kits. These images are obtained from two different litters and each image represents a different kit

To more strongly link the numerous varicosities and other abnormalities seen in the Zika positive brains with altered blood vessel morphology we used immunohistochemistry to reveal blood vessels in normal and Zika treated brains. Figure 8 shows an example of normal cortex at P0 and one infected with Zika virus, both stained with a marker for blood vessels (anti CD31). In the normal cerebral cortex the vessels have a regular morphology (Figure 8a) whereas in the Zika infected section, numerous vessels are hypertrophied and show abnormal appearance (Figure 8b, white arrows). Additional examples from other Zika treated brains can be seen in Figure 9 indicating that many blood vessels in seven different kits are hypertrophied and disorganized.

**Figure 10:**
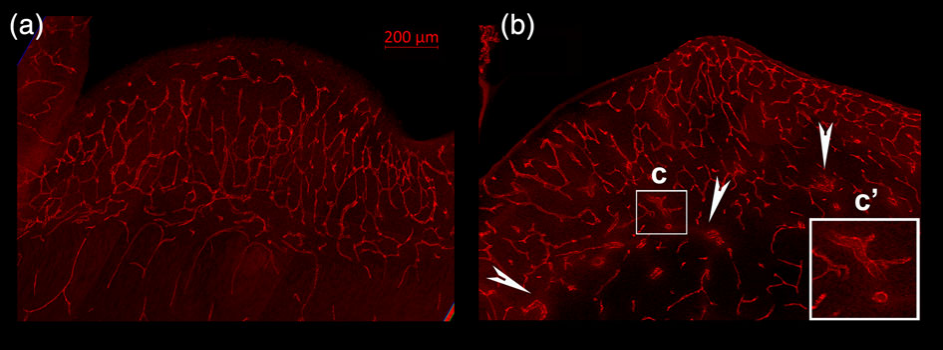
Images of blood vessels taken through the GE of a control and Zika treated animal. The control GE (a) shows blood vessels that are clearly organized and not hypertrophied. The pattern of distribution of blood vessels in the Zika treated GE (b) is disorganized, however, and also reveals hypertrophied vessels. An example is outlined with the white box (c) and shown at higher power in c’. White arrow heads also point to abnormal appearing blood vessels

To further analyze the abnormal morphology related to the caudate and GE shown in the combined Zika infected brain Trace images (Figure 8) we also visualized blood vessel immunoreactivity in the GE of control and Zika treated brains. The blood vessels in the control GE, as revealed by CD31 immunoreactivity (Figure 10a), are not hypertrophied and show a regular and evenly spaced distribution. The CD31 immunoreactivity in the GE of the Zika infected brains, however, reveal many hypertrophied vessels and an overall disorganization of the pattern of blood vessel distribution (Figure 10b). A higher power view of abnormal appearing blood vessels are outlined in the white box (Figure 10c, c’). This alteration in the blood vessel distribution may underlie the abnormalities in the MR images seen in Figure 7 in the region of the GE.

## 6 PROLIFERATIVE ZONES

**Figure 11:**
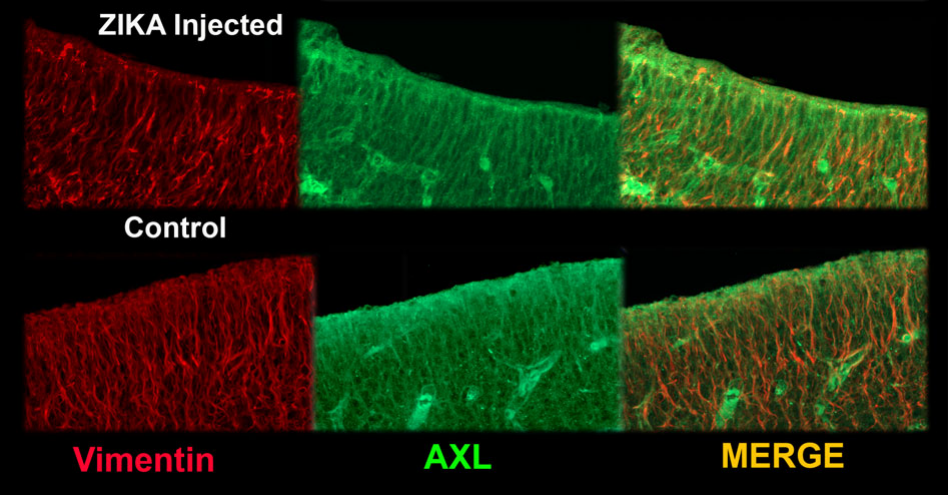
Example of AXL (green) and vimentin (red) immunoreactivity in the ventricular zone of Zika treated and control tissue. In many places, the Zika reactivity and vimentin immunostaining overlap. The overall distribution appears similar in both types of tissue

There are several presumptive entry sites for the Zika virus in the mammalian CNS. Nowakowski et al. (2016) suggested that a number of cell types in the cerebral cortex of humans and ferrets are likely sites for viral entry, including TYRO3, CD209, HAVCR1, AXL and others. They demonstrated that one of these receptor sites, AXL, is prevalent in the radial glia, astrocytes, and endothelial cells of several species, including humans, and ferrets (Nowakowski et al., 2016). We confirmed in our specimens that AXL reactivity was clearly present in the ventricular zone of normal and treated ferret kits and overlapped with vimentin immunoreactivity, which is an excellent marker for radial glia in ferrets (Juliano, Palmer, Sonty, Noctor, & Hill, 1996) (Figure 11). AXL immunoreactivity also revealed endothelial cells of presumptive blood vessels (Figure 11).

**Figure 12:**
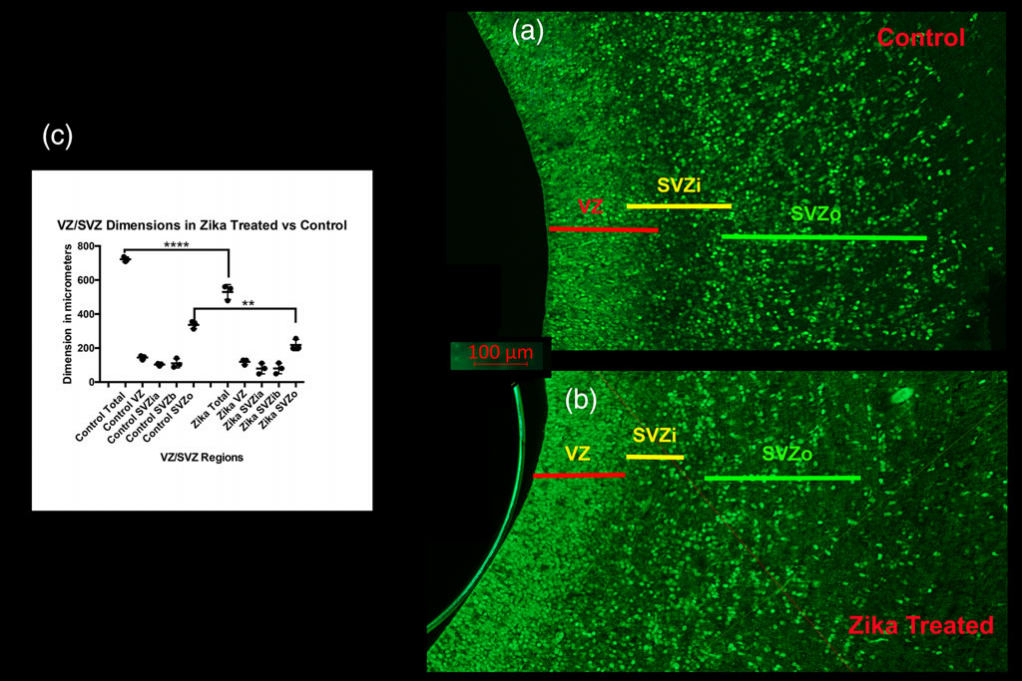
Dimension of the VZ and SVZ in control, and Zika treated tissue. (a) (control) and (b) (Zika treated) shows the extent of the ventricular zone (VZ) and the inner (SVZi) and outer (SVZo) ventricular zones in the P0 ferret. The SVZi in the Zika treated animal is less distinct than that in the normal animal. These sections are immunoreacted with SOX2, a transcription factor, which reveals neural progenitor cells. (c) Shows the measurements of each region taken for three animals in each group. This illustrates that the overall dimension of the VZ and SVZ (SVZi +SVZo) was diminished in the Zika treated animals as well as the specific dimension of the SVZo (ANOVA followed by a Tukey multiple comparison test). **** *p* < 0.0001; ** *p* < 0.001

As regions that contribute to the overall growth and proliferation of the brain may limit normal development and lead to features similar to microcephaly, we evaluated the dimensions of the VZ, and the inner and outer parts of the SVZ. To further confirm distinctions between the proliferative zones in the control and Zika treated brains, we used antibodies against SOX2 as a marker for cell renewal. Since these regions may be specifically affected by infection with Zika virus we evaluated the dimension of the VZ and the inner and outer parts of the SVZ (SVZi and SVZo) by measuring the thickness of these regions on histological sections. Measuring these areas shows that the overall extent of the VZ and all parts of the SVZ combined are significantly reduced in the Zika infected specimens (Figure 12). We also see that the SVZi is less distinct after Zika infection and that the SVZo is significantly reduced compared to control regions.

## 7 DISCUSSION

The infection of ferret jills during mid gestation resulted in kits with prominent abnormalities of brain volume, morphology, and vasculature. While these observations were in the absence of detectable viral load using blood and organ RT‐PCR measurements, both the complications of pregnancy in the infected ferrets, the identification of gross and focal MRI abnormalities, and the presence of histopathologic features in many of the Zika treated specimens suggest that the ferret is vulnerable to the effects of Zika infection during development and may provide a critical model for understanding the etiology of neurodevelopmental abnormalities that result following Zika infection in humans.

### 7.1 Variability of abnormal features

One of the more obvious findings in our study was the variability within the Zika treated litters of MRI, CT, and histologic outcomes, suggesting that the virus did not have equal access to each fetus. Ferrets often have large litters, strengthening their attractiveness as an animal model to study viral infections. It is not clear why select animals in a litter become more strongly infected than others and show different characteristics, but similar outcomes have been reported for human pregnancies in that only a fraction of women with Zika virus infections give birth to infants with microcephaly; in addition, maternal infection levels and fetal outcome may not be strongly related (Brasil, et al., 2016) (Szaba et al., 2018). A recent report by the CDC, however, suggests that babies born to infected mothers show symptoms consistent with learning disabilities as they develop over the first year or two of life. These problems are not as severe as major malformations of the brain or other body regions but in accord with impaired neural development (CDC report Aug 2018; www.cdc.gov/vitalsigns/zika-territories). In our study, we observe animals in the same litter with severe malformations, as well as those with more minor deficits. We also see those apparently relatively normal‐appearing but demonstrating skulls and brains of a small size compared to controls. The animals that we find with minor deficits may be comparable to the babies reported by the CDC that develop problems over the first year of life. In the current study, the animals are injected with the virus subcutaneously, similar to how people are usually infected. Few of the rodent studies use subcutaneous delivery, however, most likely increasing the rate of infection in the offspring by using intravenous, intraventricular, or direct brain infection routes. Studies using rodents also report variability in offspring after various methods of infection (Cugola et al., 2016; Garcez et al., 2018).

One obvious variable finding is the severe malformation in a minority of our specimens—failure of the skull to form properly as seen in Figure 2. Human observations indicate that skull malformations are common in babies born with microcephaly and possess some similarities with the gross malformations that we see in several animals (Moore et al., 2017). The sutures in the skull are not well‐formed overall in the animals that received infection with Zika, which is similar to a pattern seen in newborn infants infected with microcephaly. Garcez et al. (2018) also report poorly formed sutures in an immune incompetent mouse model.

One defect that appears relatively consistent in animals with Zika infection is the diminished size of the brain. This is especially obvious with the MRI quantification, but also seen in other measurements. Most of our analysis involved the telencephalon, and the timing of our infections corresponded with the timing of neocortical development, but other brain regions could also be reduced in size. Surprisingly, the cortical plate of infected animals seemed relatively equal in size to those in control animals, whereas regions of proliferation showed reduction in size. It may be that if we assessed these regions at different time points, more distinctions could be observed, especially as in ferrets, cortical neurons continue to be generated and migrate into the neocortex postnatally.

### 7.2 Placenta/route of infection

Although there are implicated routes for Zika infection of the brain, we do not yet know the precise route of infection. Several groups suggest the most likely route of infection is through the placenta, probably through the umbilical vein (Alvarado & Schwartz, 2017). Nowakowski and colleagues (Nowakowski et al., 2016) found that entry sites for the Zika virus are strongly represented in endothelial cells; others suggest that the Zika virus preferentially infects the umbilical vein through AXL receptors with high efficiency compared to other flaviviruses (Richard et al., 2017). The virus is also able to replicate in the placenta, creating an important site to directly infect the fetus (Green et al., 2016). This may also partially explain why we did not see a viral titer in the blood of the mother, if the placenta is an important site of Zika replication. Szaba et al. (2018)) reported that even low levels of maternal infection can cause substantial placental infection and variable effects in the fetus. In addition, each placenta may not have the same access to the virus through its individual umbilical vein. This may illuminate why in our study variable levels of infection appear to occur in different kits, each with its own placenta and umbilical vein.

Animals infected with the Zika virus in our study presented with substantial varicosities in the parenchyma of their brains. A recent article describes blood vessel pathology after Zika infection, implicating impaired angiogenesis in leading to diminished neurogenesis and microcephaly (Garcez et al., 2018). In our study, we suspected the varicosities seen in MRI might reflect abnormal vasculature and further established that immunohistochemical markers to visualize blood vessels confirm that the Zika infected animals show impaired blood vessel morphology, similar to the findings of Garcez et al. (2018). The blood vessels of infected animals are often larger in diameter and show loss of an overall patterned distribution. Compromised angiogenesis could interfere with a number of developmental features, including production and migration of neurons, leading to microcephaly. As we observed obvious varicosities in the brain using MRI, this suggests that this method of analysis may be a highly useful noninvasive method of observing pathology after infection with Zika virus. Miner et al. (2016) observed that placental infection occurred in their study after Zika infection, leading to problems with placental vasculature. Infection of the placenta is therefore an important factor that might impact the cerebral vasculature and lead to poorly formed blood vessels. We did not specifically evaluate the vasculature of the placenta in this study.

### 7.3 Timing and sites of infection

It is likely that infection with the Zika virus early during a pregnancy has a greater effect than a later infection (Brasil, et al., 2016), although we still do not have a complete knowledge of when during pregnancy the Zika virus causes fetal malformations. There is also evidence that infection during different trimesters has specific effects. For various technical reasons, in the current experiments we infected pregnant ferrets on embryonic Day 20, which corresponds to generation of the subplate, or very early during the development of the cerebral cortex (Noctor et al., 1997). This date relates to the approximate middle of the second trimester in human pregnancies. It is possible that if the virus was delivered earlier during pregnancy the results of infection would have been more severe or extensive. Nevertheless, we saw a variety of results impacting the morphology and development of the brain and skull implicating the timing of infection during the middle of the second trimester as having a strong effect on neural development. Studies in rodents find that infection even in correspondingly later periods of gestation result in severe deformities, although as suggested earlier, many of them use routes of infection that may have a more direct effect (Garcez et al., 2018).

Nowakowski et al. (2016) reported that putative entry sites for flaviviruses occur in several categories of cells in the developing brain. One of these sites, AXL, is present in several cell types that populate the brain, in addition to sites in the placenta. These include radial glia, astrocytes, and endothelial cells. As radial glia are neural progenitors, this provides the virus direct ability to impair cells that populate the developing cerebral cortex. Chavali et al. (2017) recently reported that the RNA of Zika virus binds to a specific RNA binding protein (Mushashi), which is strongly expressed in radial glial cells. Several studies in rodents and culture models find that progenitor cells are affected after Zika infection and show reduced neural proliferation and neuronal death in the affected brains or cultures (Wen, Song, & Ming, 2017 for review). Additional work reports specific reduction of the thickness of the cerebral cortex and numbers of neural progenitor cells (Garcez et al., 2018; Song et al., 2017). Although we see a reduction in proliferative zones, we do not see a substantial reduction in thickness of the cerebral cortex at P0, whereas several of the rodent studies do find reduced neocortical thickness. We find a global reduction in brain volume in the infected brains, as revealed by our MRI studies, also suggesting that cell proliferation reduces after Zika virus infection. We also observe a decrease in the thickness of zones that contain neural progenitor cells as well as overall reduction in brain volume. Substantial evidence suggests that infection with Zika virus finds its way to the placenta via the umbilical vein and accesses the fetus via the vasculature. Neural progenitor cells appear especially susceptible to viral attack and demonstrate cell death in culture and in vivo studies (Ming, Song, & Tang, 2017).

Our observation that the proliferative region surrounding the GE contains extensive lesions not seen the same extent in the neocortical ventricular zone, suggests that inhibitory cells and their migration into the developing cortical plate may be more involved than excitatory cells. In this study we did not specifically assess the distribution of inhibitory versus excitatory cells the neocortex, but can follow up on this possibility in the future. Again, this particular type of involvement may reflect the timing of the Zika injection; other regions might be more seriously implicated if the infection occurred either earlier or later.

### 7.4 Animal models

Studies of Zika virus infection generally use rodents and primates. A number of experiments also use cell culture including direct infection of various types of cells and 3D culture models (Wen et al., 2017). Although rodents can develop signs of infection, they usually do not show the severe types of disease found in people and their offspring. Because of the importance of developing an animal model demonstrating the types of congenital malformations that occur after human infection, researchers turned to direct infection of the brain, or to direct infection of young developing animals as well as immune deficient animals, or to blockade of immune receptors (Krause, Azouz, Shin, & Kumar, 2017; Smith, 2017). This approach allows study of the effects of Zika pathology in intact animals and during pregnancy. Although multiple studies using immune deficient animals reveal substantial information in understanding the mechanism and etiology of this disease, it is not ideal to use animals that lack specific mechanisms to fight viral infections. Primate studies are important to analyze this viral infection, but it is not practical to use them for large scale studies. The ferret, therefore, makes an excellent model to study the effects of Zika virus. As an animal with a gyrencephalic cortex and a greater distinction between zones of proliferation during development, it can be an important model to further study the effects of infection with Zika virus.

### 7.5 Conclusion

It is problematic that we were not able to show levels of viremia in the blood of the ferret jills at different times post infection. It is possible that we missed the proper time to find the virus in the blood after infection, although our samples included the times that usually captured infection levels. The ferret kits show obvious signs of infection and our quantitative observations are encouraging, suggesting that although we do not see evidence in the blood of Zika infection, there may be reasons why. As indicated, we may have missed the proper timing to observe viral infection in the blood, or the infection may show increased proliferation in a region that we did not test, such as the placenta. Overall, we demonstrate that the ferret is an excellent model to study the effects of Zika virus infection in the brain. Our finding of variability in infection is an important outcome that needs further analysis to determine why certain embryos are seriously infected and others are spared.

## Supporting information

Supplemental Figure 1 Videos of 3D CT reconstructions of skulls from a control litter. Although there is some variability in the skull dimension, they are similar in size.

Supplemental Figure 2 Videos of 3D CT reconstructions of skulls from a litter that received an infection with Zika virus. Substantial variability can be seen in the dimension of the skulls. Those indicated with an asterisk were smaller than the others.
