# Supplementary material for: The effect of Zika virus infection in the ferret
Source: J Comp Neurol. 2019 Feb 15;527(10):1706–19. doi: 10.1002/cne.24640 (PMC6593673; doi:10.1002/cne.24640)
Supplement: Supplementary file 1 — Supplemental Figure 1 Videos of 3D CT reconstructions of skulls from a control litter. Although there is some variability in the skull dimension, they are similar in size. [file CNE-527-1706-s001.pptx]

## Slide 1
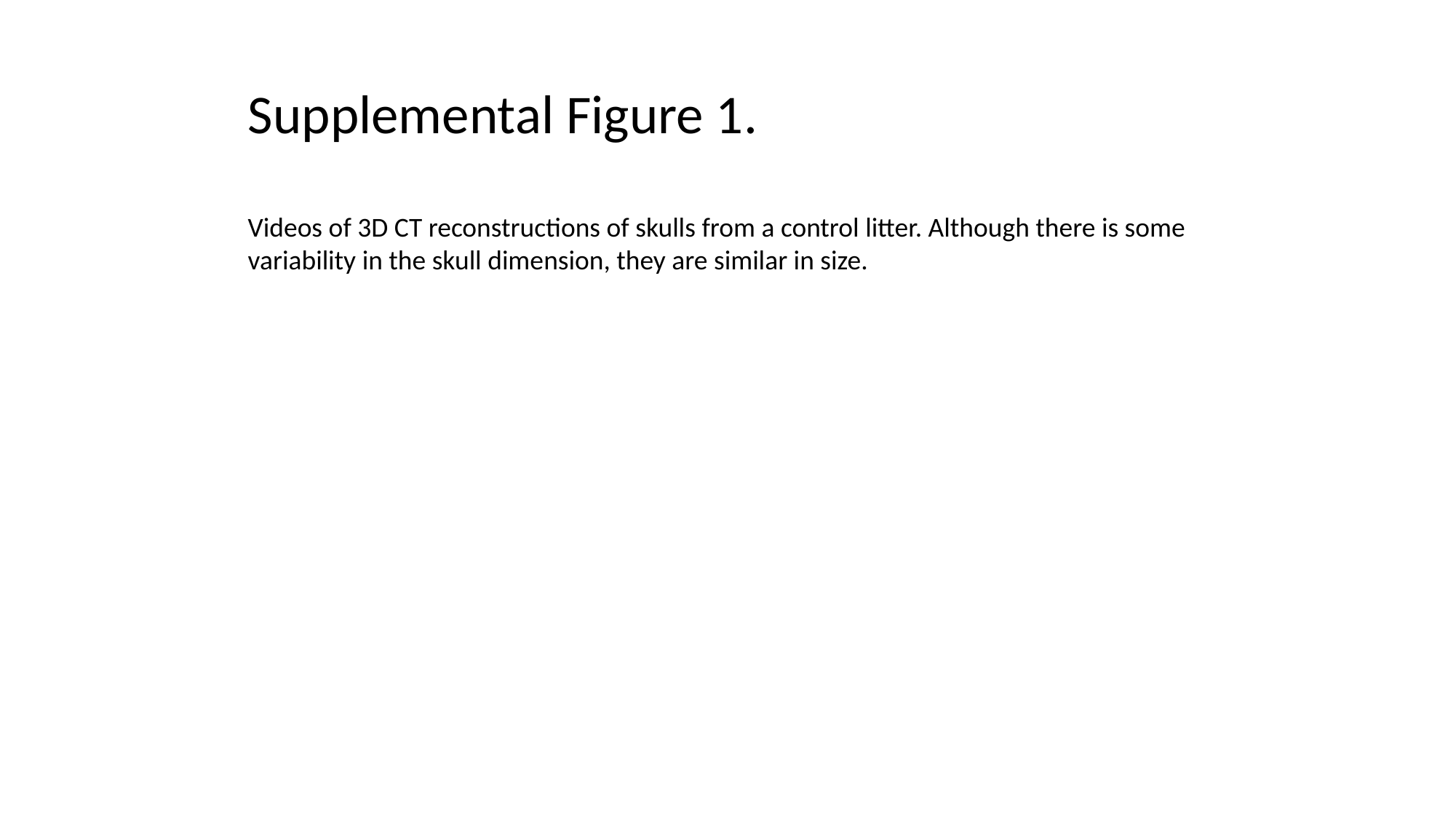

Supplemental Figure 1.
Videos of 3D CT reconstructions of skulls from a control litter. Although there is some variability in the skull dimension, they are similar in size.

## Slide 2
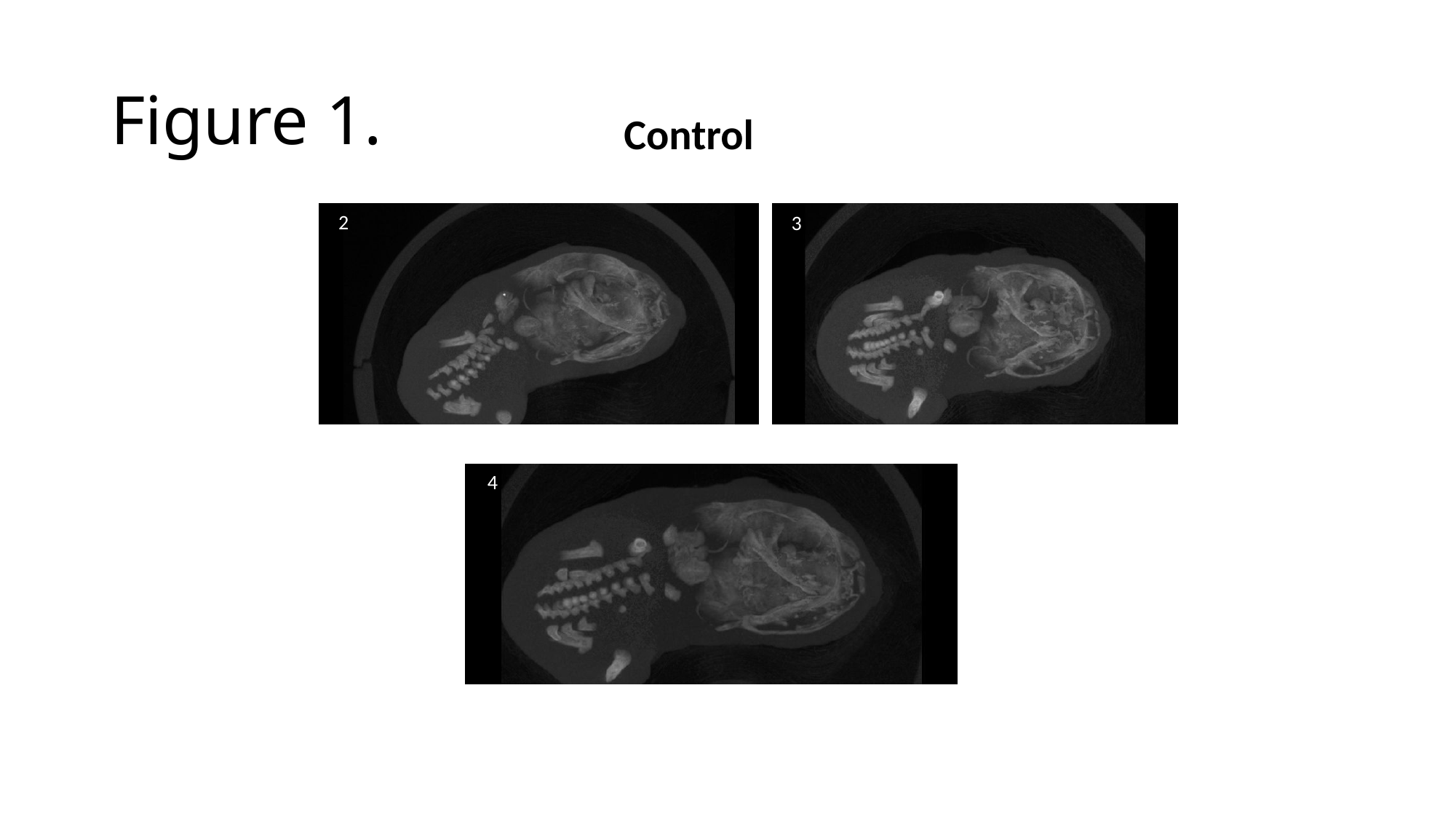

# Figure 1.
Control
2
3
4
