# Supplementary material for: The effect of Zika virus infection in the ferret
Source: J Comp Neurol. 2019 Feb 15;527(10):1706–19. doi: 10.1002/cne.24640 (PMC6593673; doi:10.1002/cne.24640)
Supplement: Supplementary file 2 — Supplemental Figure 2 Videos of 3D CT reconstructions of skulls from a litter that received an infection with Zika virus. Substantial variability can be seen in the dimension of the skulls. Those indicated with an asterisk were smaller than the others. [file CNE-527-1706-s002.pptx]

## Slide 1
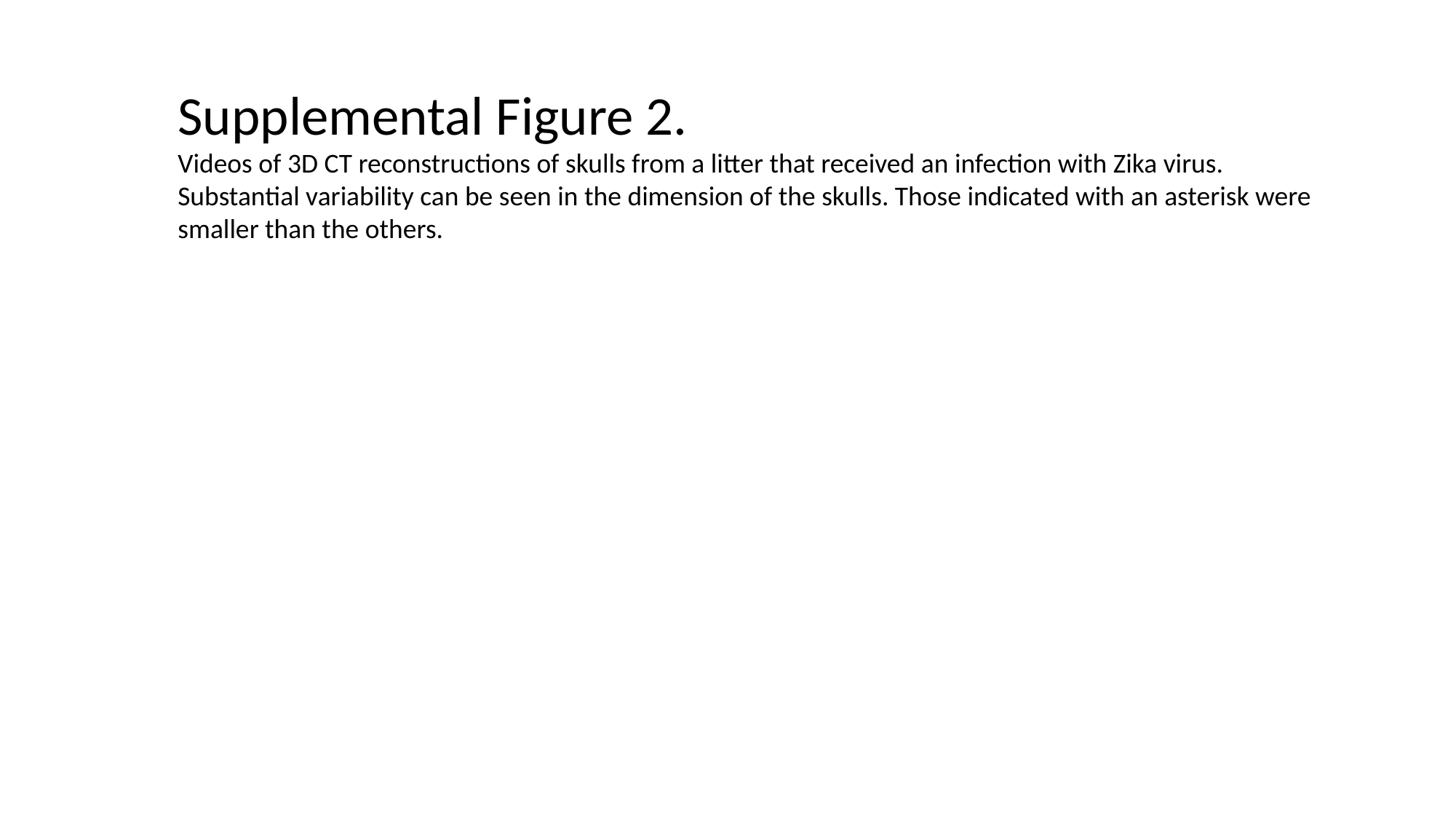

Supplemental Figure 2.
Videos of 3D CT reconstructions of skulls from a litter that received an infection with Zika virus. Substantial variability can be seen in the dimension of the skulls. Those indicated with an asterisk were smaller than the others.

## Slide 2
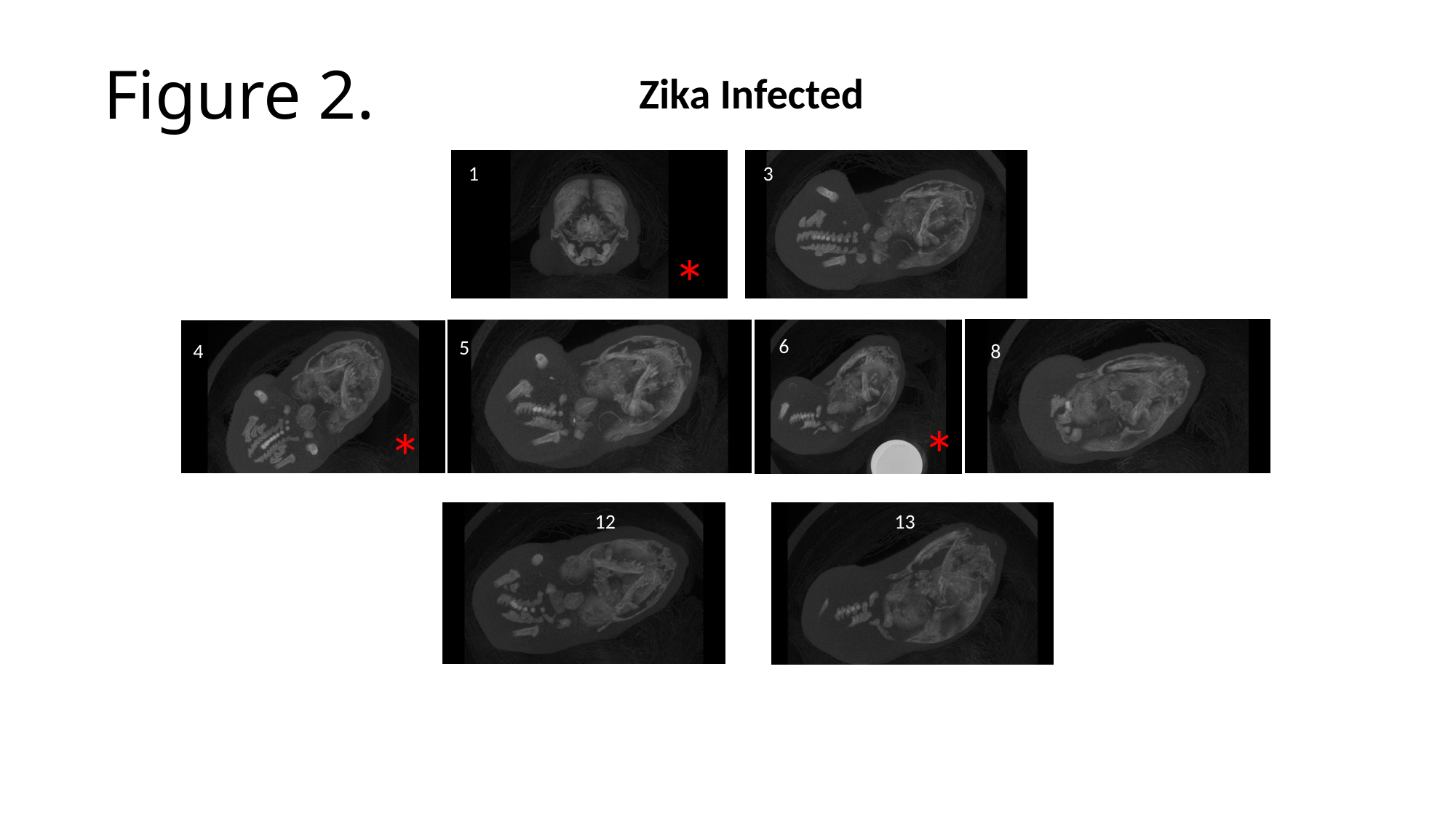

# Figure 2.
Zika Infected
1
3
*
6
5
4
8
*
*
10
12
13
